# Patterns of toxicity burden for FDA-approved immune checkpoint inhibitors in the United States

**DOI:** 10.1186/s13046-022-02568-y

**Published:** 2023-01-05

**Authors:** Fan Yang, Chloe Shay, Marin Abousaud, Chris Tang, Yamin Li, Zhaohui Qin, Nabil F. Saba, Yong Teng

**Affiliations:** 1 Department of Hematology and Medical Oncology, Winship Cancer Institute, Emory University School of Medicine, 201 Dowman Dr, Atlanta, GA 30322 USA; 2 Wallace H. Coulter Department of Biomedical Engineering Georgia Institute of Technology and Emory University, Atlanta, GA 30322 USA; 3 Department of Pharmaceutical Sciences, Emory Healthcare, Atlanta, GA 30322 USA; 4 Department of Pharmacology, SUNY Upstate Medical University, Syracuse, NY 13210 USA; 5 Department of Biostatistics and Bioinformatics, Rollins School of Public Health, Emory University, Atlanta, GA 30322 USA

**Keywords:** immune checkpoint inhibitors, Immunotherapy, Adverse events, Toxicity burden, Therapeutic intervention

## Abstract

**Background:**

Immune-related adverse events (irAEs) are a common phenomenon in cancer patients treated with immune checkpoint inhibitors (ICIs). Surprisingly, the toxicity burdens of these irAEs have not been illustrated clearly. In this study, we analyzed irAEs for seven FDA-approved ICIs in cancer treatment to show the pattern of toxicity burden among cancer patients.

**Methods:**

irAEs associated with seven FDA-approved ICIs, including three PD-1 inhibitors (cemiplimab, nivolumab and pembrolizumab), three PD-L1 inhibitors (atezolizumab, avelumab and durvalumab), and one CTLA-4 inhibitor (ipilimumab), were analyzed based on data from 149,303 reported cases (from January 1, 2015 to June 30, 2022) collected from the FDA Adverse Events Reporting System (FAERS) public dashboard. Proportions of serious irAEs and correlations with tumor type, age and sex were assessed via R package and GraphPad software.

**Results:**

irAEs related to anti-PD-1 ICIs required less hospital care resources compared with anti-PD-L1 and anti-CTLA-4 ICIs. Patients treated with pembrolizumab had relatively fewer serious cases. Treatment with ICIs led to the highest probability of serious irAEs in patients with lung cancer. ‘Respiratory, thoracic and mediastinal disorders’ and ‘gastrointestinal disorders’ were the two most common groups of disorders caused by the seven ICIs studied. ‘Cardiac disorders’ was the main type of disorders caused by these ICIs in cancer patients aged 65–85, while ‘reproductive system and breast disease’ was the main type of disorder in cancer patients aged 18–64. ‘Respiratory, thoracic, mediastinal diseases’ and ‘reproductive system and breast diseases’ were the main types of disorders associated with treatment with these ICIs in male and female patients, respectively.

**Conclusion:**

Tissue and organ toxicities of ICIs are age and sex specific. There are risks of respiratory and urinary system toxicity in male patients and reproductive system toxicity in female patients treated with the ICIs studied. Future studies on the toxicity burden of ICIs should incorporate age and sex differences to better understand the relevance of ICI toxicity burden to human immune function to develop appropriate tumor immune and therapeutic intervention strategies.

**Supplementary Information:**

The online version contains supplementary material available at 10.1186/s13046-022-02568-y.

## Background

Immune checkpoint inhibitors (ICIs) have revolutionized the treatment landscape for multiple cancers, demonstrating effective and durable responses and becoming the standard of care for a variety of malignancies [1]. The Food and Drug Administration (FDA) has approved various ICIs for cancer therapy [2], including programmed death-1 (PD-1) receptor inhibitors (e.g., cemiplimab, nivolumab and pembrolizumab), programmed death ligand-1 (PD-L1) inhibitors (e.g., atezolizumab, avelumab and durvalumab), and cytotoxic T lymphocyte-associated antigen (CTLA-4) inhibitors (ipilimumab) [3–5]. Although these ICIs improve patient outcomes in various clinical settings, they pose concomitant risks of immune-related adverse events (irAEs) [6]. These irAEs are unique, delayed, and long-lasting, and can involve any tissue or organ system [7]. Given the increasing use of ICIs, there has been an increase in the burden of both clinical and financial toxicity. More common irAEs include skin toxicities, colitis, hepatitis, pneumonitis, nephritis, and endocrinopathies (i.e. thyroid abnormalities) [8]. Rare irAEs can have unique clinical presentations that can pose a serious issue if not identified promptly, such as encephalitis, myocarditis, and hematologic toxicities (i.e. hemolytic uremic syndrome). From a financial toxicity standpoint, patients, family members, healthcare systems, and insurance companies experience the significant economic weight of ICI treatment and irAEs [9]. Factors such as increased utilization of ICIs due to their expanding FDA approvals in several cancers, high drug expenses with high out-of-pocket expenditures, and costs associated with managing irAEs (e.g., hospitalizations and the use of biologic agents) all contribute to the exponentially increasing cost of cancer care with ICIs. As ICI prevalence continues to increase with immunotherapy being introduced into earlier stages of disease (neoadjuvant and adjuvant settings) and more combinations with ICIs being developed, clinical and financial toxicity will only become more problematic.

irAEs can range widely in severity from mild (grade 1) to life-threatening (grade 4) [2, 6, 7]. By targeting immune checkpoints, ICI-associated irAEs, characterized by T-cell infiltration to a number of organ systems, can occur [6, 10, 11]. The physical burden of irAEs is significant, as they can lead to hospitalizations, long term use of high dose steroids which have several AEs (e.g., hyperglycemia, increased risk for infections and bone loss/osteoporosis), or even permanent discontinuation of ICIs. In solid tumor patients, the incidence of any-grade irAEs in trials is 66% with PD-1/PD-L1 inhibitor monotherapy and 72% with ipilimumab monotherapy [4, 12]. Combined PD-1 and CTLA-4 blockade results in considerably higher rates of irAEs in comparison to anti-PD-1 alone (55%-60% vs 10%-20% high-grade events) [5, 13–15]. A retrospective meta-analysis conducted by Wang et al. reported immunotherapy toxicity-related fatality rates of 0.36% with anti-PD-1, 0.38% with anti-PD-L1, 1.08% with anti-CTLA-4, and 1.23% with combined anti-PD-1/anti-PD-L1 and anti-CTLA-4 [12]. The type of fatal irAEs varied depending on the regimen; the most common fatal irAE with anti-CTLA-4 treatment was colitis (70%), whereas the most common fatal irAEs with anti-PD-1/anti-PD-L1 treatment were pneumonitis (35%), hepatitis (22%), and neurotoxicity (15%). For combined anti-PD-1/anti-PD-L1 and anti-CTLA-4 treatment, the most common fatal irAEs were colitis (37%) and myocarditis (25%) [12]. Furthermore, ICIs have the potential to trigger immune-related endocrine diseases in tumor patients, such as thyroid and pituitary dysfunction, and these complications are relatively more frequent than expected (e.g., 11.8% in anti-PD-1 treatment, 13.4% in anti-PD-L1 treatment, 5% in anti-CTLA4 treatment, and 18.5% in sequential and/or combination treatment) [16–19].

Interrogating irAEs in cancer patients remains to be evaluated systemically. In the present study, we estimated the toxicity burden of seven FDA-approved ICIs by analyzing irAEs among treated patients in the United States. The proportions of total and serious irAEs for each ICI and the proportion of serious irAEs for each ICI by cancer type were calculated. Correlations between tissue or organ disease and patient demographics were also calculated for each ICI. Our comprehensive assessment of irAEs according to the latest data reported by the FDA summarizes the major types of risk factors correlated with irAEs, providing a reference for clinicians to predict the occurrence of irAEs resulting in a timely process in clinical practice.

## Methods

### Cases of irAEs associated with ICI treatment

All cases for this study were obtained from the FDA Adverse Events Reporting System (FAERS) public dashboard (https://www.fda.gov/drugs/questions-and-answers-fdas-adverse-event-reporting-system-faers/fda-adverse-event-reporting-system-faers-public-dashboard), and a total of 149,303 cases from January 1, 2015 to June 30, 2022 were analyzed for seven FDA-approved ICIs, including three PD-1 inhibitors (cemiplimab: 492 cases, nivolumab: 60,469 cases, and pembrolizumab: 34,962 cases), three PD-L1 inhibitors (atezolizumab: 16,117 cases, avelumab: 2,136 cases, and durvalumab: 6,974 cases), and one CTLA-4 inhibitor (ipilimumab: 28,153 cases). Serious AEs (cemiplimab: 472 cases; nivolumab: 55,027 cases; pembrolizumab: 29,379 cases; atezolizumab: 15,309 cases; avelumab: 1,898 cases; durvalumab: 6,486 cases; and ipilimumab: 24,675 cases) and deaths associated with these ICIs were downloaded and counted.

### Proportional reporting ratio (PRR) analysis

A PRR was used to analyze the irAEs via the Chi-squared test, and the odds ratio (OR) was calculated for irAEs associated with each drug using retrospective case–control studies. First, all cases were divided into seven outcome groups (died, disabled, hospitalized, life-threatening, non-serious, required intervention, and other outcomes) depending on the MedDRA dictionary Preferred Term (PT) and the percentage of cases in each group was counted using R software. The ratios of total serious cases and deaths were calculated for each ICI. Then we focused on irAEs for each drug in each individual cancer type and counted the value for each tumor type via R software, including bladder cancer, breast cancer, colorectal cancer, endometrial cancer, glioma, head and neck cancer (HNC), hepatocellular carcinoma, lung cancer, melanoma, ovarian cancer, prostate cancer, renal cancer, and thyroid cancer. To assess the tumor-type-specificity of irAEs, correlations between irAEs and age (excluding 0–3 years old) and sex were evaluated for each ICI using R software in 18 tissues or organs (reproductive system and breast, cardiac, musculoskeletal and connective tissue, ear and urinary, respiratory, thoracic and mediastinal, renal and urinary, endocrine, skin and subcutaneous tissue, blood and lymphatic system, psychiatric, immune system, eye, hepatobiliary, gastrointestinal, nervous system, vascular, metabolism and nutrition, and neoplasms benign, malignant and unspecified). All ‘tissue or organ disorders’, as the one of the ‘reaction groups’, were defined by the Medical Dictionary for Regulatory Activities (MedDRA) (https://fis.fda.gov/sense).

### Tissue-specific gene expression analysis

To attempt to evaluate irAEs with tumor-type specificity at the gene expression level, the expression levels of PD-1, PD-L1, and CTLA-4 in various tissues or organs (such as brain, heart, lung and skin) from male and female patients were analyzed in the GTEx portal (https://gtexportal.org/home/gene/). Gene expression levels were normalized using log10 (TMP + 1).

### Statistics

Chi-squared test and case–control studies were used to evaluate irAEs associated with the seven drugs. An OR value > 1 indicated that the drug is a positive factor for irAEs, while OR value < 1 indicated the drug is a negative factor. *P* < 0.05 represented a statistically significant difference. Data statistics and correlation analysis were performed using the GraphPad prism 9 and R packages (Rmisc, corrplot, ggcorrplot, grDevices and vegan).

## Results

### irAEs related to pembrolizumab require less hospital care resources and are associated with relatively fewer serious cases

**Fig. 1:**
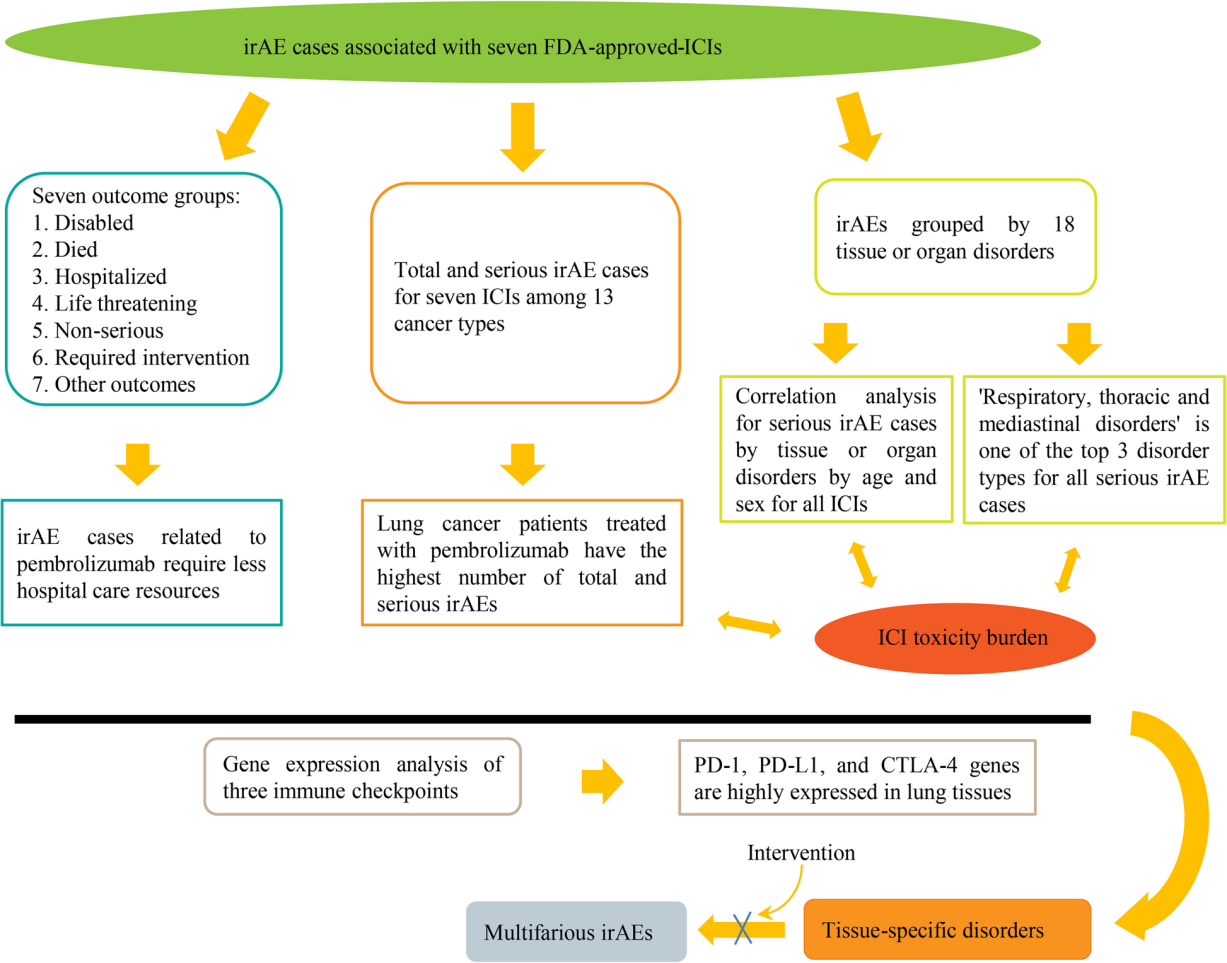
Flow chart of irAE studies for seven FDA-approved ICIs**:** ICI toxicity burden causing tissue or organ disorders may exacerbate ICI-associated irAEs

**Fig. 2:**
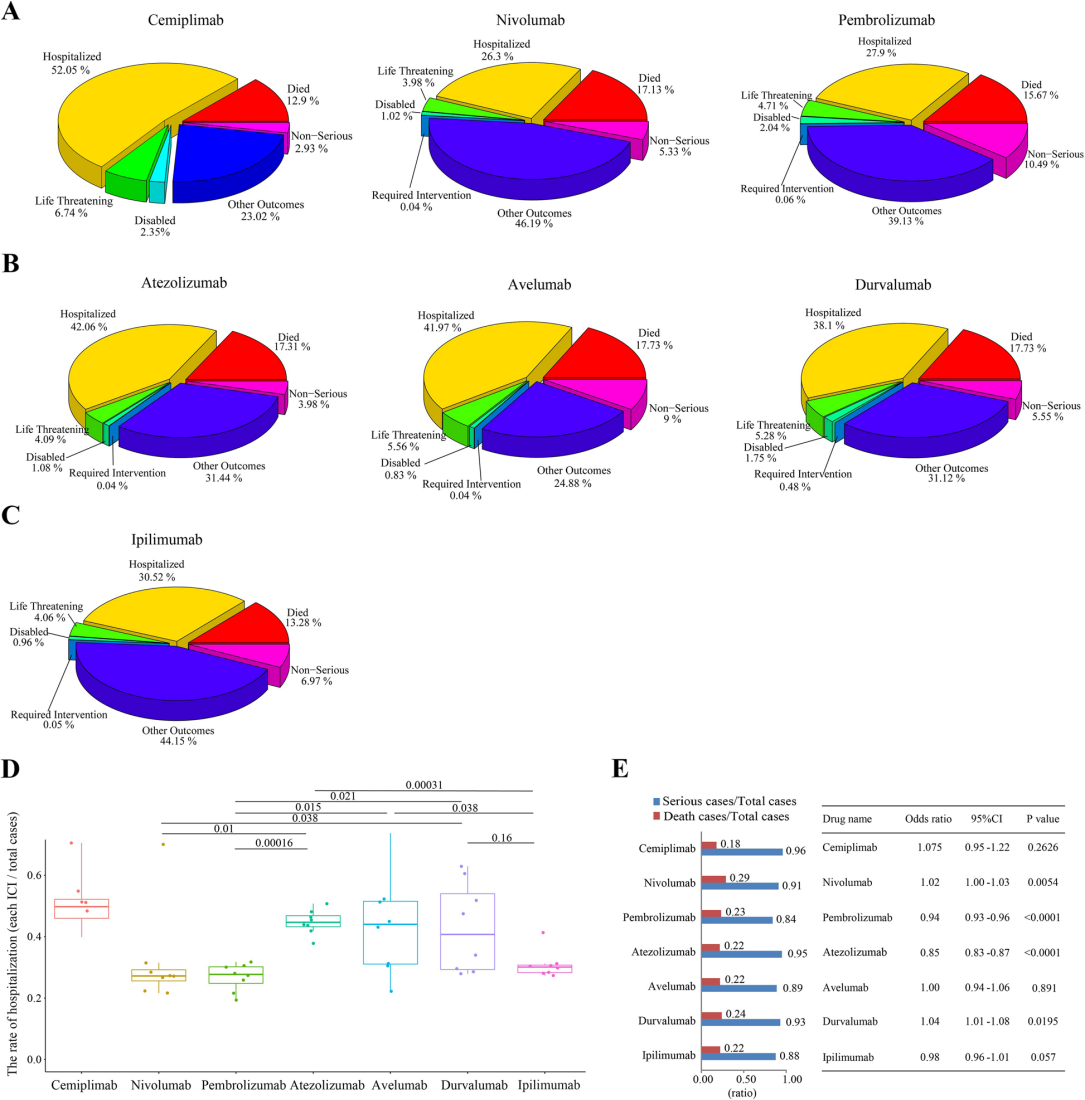
Distribution of irAE cases among patients treated with seven FDA-approved ICIs according to the FDA dataset in the past seven years. **A**-**C** irAE cases for each ICI were divided into seven outcome groups, including died, disabled, hospitalized, life-threatening, non-serious, required intervention and other outcomes. The total percentage for each outcome group is indicated for ICIs targeting PD1, PDL1 and CTLA4 in (**A**), (**B**) and (**C**), respectively. **D** Comparison of the rate of hospitalization among patients treated with each ICI. **E** Proportions of serious irAEs or deaths for each FDA-approved ICI in the FDA dataset (left) and summary of statistical analysis of odds ratio (OR) for seven FDA-approved ICIs (right)

Referring to the FDA dataset, all annual irAE cases for each drug were divided into the 7 outcome groups separately (died, disabled, hospitalized, life-threatening, non-serious, required intervention, and other outcomes) (Fig. 1). Here, we calculated the proportion of each outcome group for each individual ICI per year (Supplementary Figure S1). To assess the weight of the individual groups for each ICI, we calculated the average probability of each group for each single drug (Fig. 2A-C) and found for all drugs that hospitalization was the most common outcome (except for the “other outcomes” group). The mean rate of hospitalization varied widely among patients treated with PD-1 inhibitors and experiencing irAEs: for instance, the average hospitalization rate for cemiplimab patients was 52.05% (from 48.42% to 70.59%) and was the highest among all irAE cases associated with the seven ICIs, while that for nivolumab was the lowest at 26.3% (21.66%—31.47%) and for pembrolizumab was the second lowest at 27.9% (21.6%—31.77%). The mean hospitalization proportion for the other four ICIs ranged 30% to 45%. The proportion of hospitalizations due to irAEs was significantly lower among patients treated with anti-PD-1 ICIs than with anti-PD-L1 or anti-CTLA4 ICIs (*P* < 0.05) (Fig. 2D). Therefore, irAEs related to nivolumab and pembrolizumab require less hospital care resources.

To accurately evaluate the severity of irAEs, the proportion of serious irAEs (serious irAEs cases / total irAEs cases) and deaths (deaths / total irAEs cases) was calculated for each drug. All drugs were associated with a high proportion of serious irAEs (cemiplimab, 0.96; nivolumab, 0.91; pembrolizumab, 0.84; atezolizumab, 0.95; avelumab, 0.89; durvalumab, 0.93; and ipilimumab, 0.88), with pembrolizumab having the lowest proportion of serious irAE cases among all seven drugs (Fig. 2E). Next, we calculated the odds ratio value for these seven drugs, and found the OR values for pembrolizumab, atezolizumab and ipilimumab were 0.94 (*P* < 0.0001), 0.85 (*P* < 0.0001) and 0.98 (*p* = 0.57), respectively, while the OR for avelumab was 1 and other three drugs had ORs > 1. However, we noticed that the OR for atezolizumab was less than 1 but its irAEs ratio was high (0.95), and the OR for ipilimumab was 0.98 (< 1) but was not significant (*P* = 0.057 > 0.05).

### Serious irAEs are most common among patients with lung cancer

**Fig. 3:**
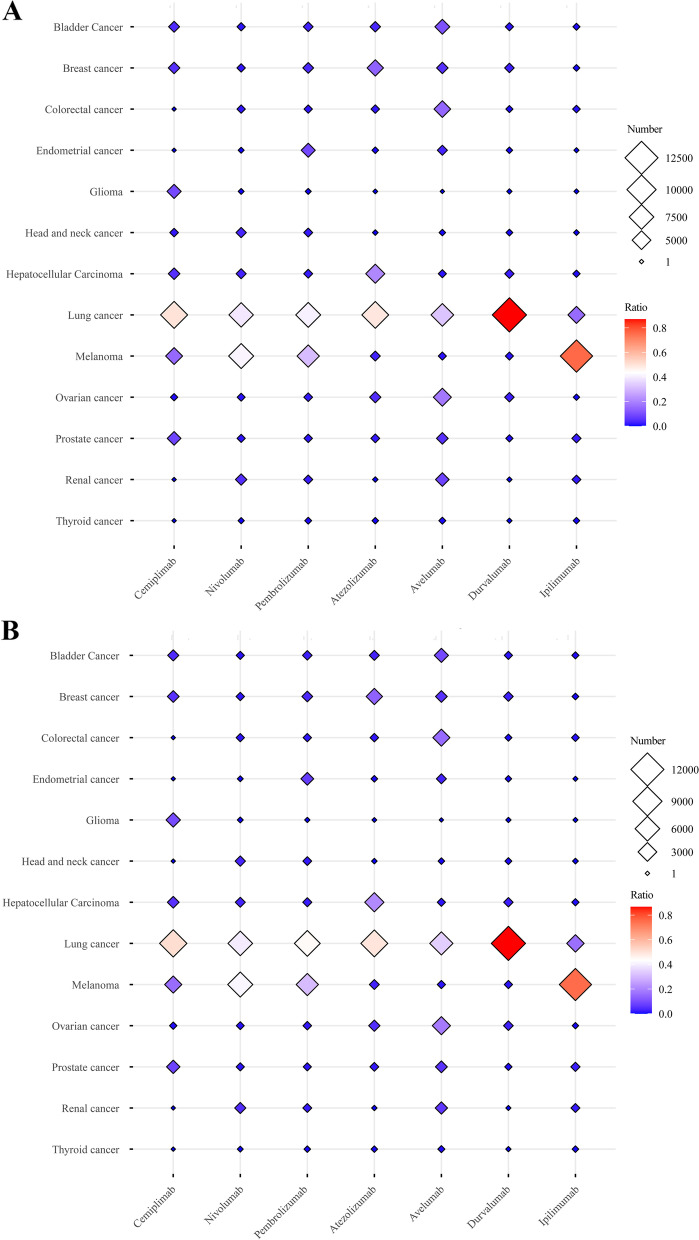
Total irAE cases and serious irAE cases for seven FDA-approved ICIs among various cancer types. **A** Total number of irAE cases for seven FDA-approved ICIs: patients with lung cancer ranked first or second for the highest number of total irAEs. **B** Number of serious irAEs for seven FDA-approved ICIs: patients with lung cancer also ranked first or second for serious irAEs

To evaluate which cancer type was most prone to irAEs with the seven drugs, 13 main cancer types were selected. First, the numbers of total and serious irAEs for each drug were calculated for each cancer type (Table 1 and Fig. 3A and B). For cemiplimab, the numbers of total irAEs (59 cases) and serious irAEs (58 cases) were higher in lung cancer patients than in the other 12 cancers, and the proportion of serious irAEs was 50% among all 13 cancer types (Supplementary Figure S2A). For nivolumab, melanoma (11,602 cases) and lung cancer (11,052 cases) patients were most prone to serious irAEs, with an incidence of 41% and 31%, respectively. Similarly, total and serious irAEs were most common among lung cancer (4,399 cases) and melanoma (6,335 cases) patients treated with pembrolizumab, at 41% and 39%, respectively. For all three PD-L1 inhibitors, the numbers of total and serious irAEs were higher among patients with lung cancer than the other 12 cancers: atezolizumab (49%), avelumab (33%) and durvalumab (87%) (Supplementary Figure S2B). From Fig. 3 and S2C (yellow), we also found that serious irAEs were most common among melanoma (76%) and lung cancer (16%) patients treated with ipilimumab among all 13 cancer types. In summary, patients are most likely to develop serious irAEs with the seven ICIs studied when treated for lung cancer.

**Table 1:** Total irAE cases and serious irAE cases for FDA-approved ICIs among 13 common tumor types

		Bladder cancer	Breast cancer	Colorectal cancer	Endometrial cancer	Glioma	Head and neck cancer	Hepatocellular carcinoma	Lung cancer	Melanoma	Ovarian cancer	Prostate cancer	Renal cancer	Thyroid cancer	Total number
		Number /ratio	Number /ratio	Number /ratio	Number /ratio	Number /ratio	Number /ratio	Number /ratio	Number ratio	Number /ratio	Number /ratio	Number /ratio	Number /ratio	Number /ratio	
Cemiplimab	Total irAE cases	5/ 0.0424	6/ 0.0508	0/ 0.0000	0/ 0.0000	11/ 0.0932	2/ 0.0169	6/ 0.0508	59/ 0.5000	18/ 0.1525	1/ 0.0084	10/ 0.0847	0/ 0.0000	0/ 0.0000	118
	Serious irAE cases	5/ 0.0438	6/ 0.0526	0/ 0.0000	0/0.0000	11/ 0.0964	0/ 0.0000	6/ 0.0526	58/ 0.5087	18/ 0.1578	1/ 0.0087	9/ 0.0789	0/ 0.0000	0/ 0.0000	114
Nivolumab	Total irAE cases	423/ 0.015	454/ 0.016	435/ 0.0153	72/ 0.0025	64/ 0.0023	966/ 0.0340	886/ 0.0312	11,130/0.3914	11,796/ 0.4149	356/ 0.0125	404/ 0.0142	1,351/ 0.0475	96/ 0.0034	28,433
	Serious irAE cases	387/ 0.014	444/ 0.0159	413/ 0.0148	64/ 0.0023	62/ 0.0022	964/ 0.0346	845/0.0303	11,052/ 0.3967	11,602/ 0.4164	337/ 0.0121	391/ 0.0140	1237/ 0.0444	65/ 0.0023	27,863
Pembrolizumab	Total irAE cases	467/ 0.0283	648/ 0.0392	250/ 0.0151	1,499/ 0.0908	42/ 0.0025	330/ 0.0200	330/ 0.0200	6,765/ 0.4101	5,154/ 0.3124	304/ 0.0184	259/ 0.0157	347/ 0.0210	101/ 0.0061	16,496
	Serious irAE cases	367/ 0.0258	555/0.0390	220/ 0.0154	979/ 0.0688	12/ 0.0008	270/ 0.0190	304/ 0.0213	6,335/ 0.4454	4,399/ 0.3093	241/ 0.0169	195/ 0.0137	282/ 0.0198	64/ 0.0045	14,223
Atezolizumab	Total irAE cases	365/ 0.0360	1,415/ 0.1396	167/ 0.0165	50/ 0.0049	2/ 0.0002	15/ 0.0015	2,123/ 0.2095	4,976/ 0.4910	306/ 0.0302	462/ 0.0456	195/ 0.0192	12/ 0.0012	45/ 0.0044	10,133
	Serious irAE cases	301/ 0.0307	1,360/ 0.1388	163/ 0.0166	49/ 0.0050	2/ 0.0002	14/ 0.0014	2,095/ 0.2138	4,829/ 0.4928	289/ 0.0295	453/ 0.0462	189/ 0.0193	11/ 0.0011	43/ 0.0044	97,98
Avelumab	Total irAE cases	49/ 0.1077	22/ 0.0490	66/ 0.1470	14/ 0.0311	0/ 0.0000	2/ 0.0045	6/ 0.0134	146/ 0.3251	6/ 0.0134	81/ 0.1804	23/ 0.0512	37/ 0.0824	3/ 0.0067	455
	Serious irAE cases	40/ 0.0930	22/ 0.0519	66/ 0.1557	13/ 0.0307	0/ 0.0000	2/ 0.0047	6/ 0.0141	145/ 0.3420	6/ 0.0142	78/ 0.1839	23/ 0.0542	26/ 0.0613	3/ 0.0071	430
Durvalumab	Total irAE cases	57/ 0.0145	102/ 0.0259	28/ 0.0071	17/ 0.0043	3/ 0.0008	23/ 0.0058	92/ 0.0234	3,427/ 0.8700	53/ 0.0135	100/ 0.0254	31/ 0.0079	3/ 0.0007	3/ 0.0008	3,939
	Serious irAE cases	52/ 0.0139	99/ 0.0265	26/ 0.0070	17/ 0.0046	3/ 0.0008	23/ 0.0062	91/ 0.0243	3,235/ 0.8666	53/ 0.0142	99/ 0.0265	30/ 0.0080	2/ 0.0005	3/ 0.0008	3,733
Ipilimumab	Total irAE cases	120/ 0.0070	90/ 0.0052	178/ 0.0105	10/ 0.0006	7/ 0.0004	33/ 0.0019	143/ 0.0084	2,660/ 0.1563	12,924/ 0.7593	66/ 0.0039	379/ 0.0222	333/ 0.0196	77/ 0.0045	17,020
	Serious irAE cases	115/ 0.0071	87/ 0.0054	171/ 0.0106	8/ 0.0005	7/ 0.0004	31/ 0.0019	131/ 0.0081	2,645/ 0.1646	12,050/ 0.7497	63/ 0.0039	369/ 0.0230	321/ 0.0200	75/ 0.0047	16,073

### FDA-approved ICIs have greatest potential to induce ‘respiratory, thoracic and mediastinal disorders’ and ‘gastrointestinal disorders’

**Fig. 4:**
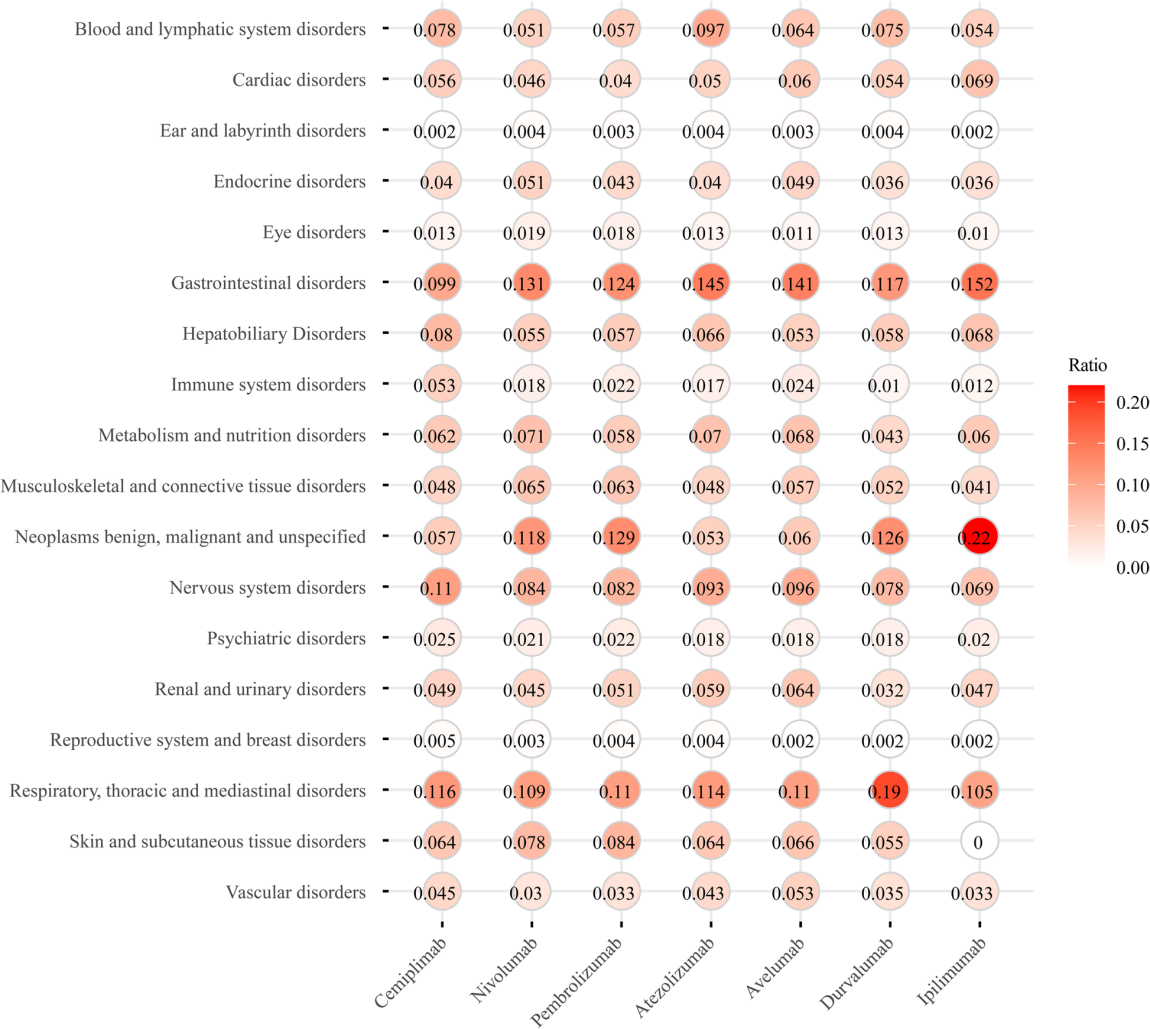
Number of irAEs grouped by 18 tissue or organ disorders for FDA-approved ICIs. Nivolumab, pembrolizumab and atezolizumab had the highest case numbers of serious irAEs. “Respiratory, thoracic and mediastinal disorders” was consistently among the top 3 for each ICI

To understand why these ICIs lead to high rates of irAEs for specific cancer types, irAEs associated with each drug were grouped according to disorders of 18 tissues or organs (Table 2, Fig. 4 and Supplementary Figure S3). For the three PD-1 inhibitors, we found the three most common tissue or organ disorders among patients treated with cemiplimab were ‘respiratory, thoracic and mediastinal disorders’ (73 cases and 11.6% of the total), ‘nervous system disorders’ (69 cases, 10.99%) and ‘gastrointestinal disorders’ (62 cases, 9.87%) (Figure S3A); for nivolumab ‘gastrointestinal disorders’ (11,231 cases, 13.14%), ‘neoplasms benign, malignant and unspecified’ (10,113 cases, 11.83%) and ‘respiratory, thoracic and mediastinal disorders’ (9,317 cases, 10.90%); and for pembrolizumab ‘gastrointestinal disorders’ (6,764 cases, 12.92%), ‘neoplasms benign, malignant and unspecified’ (6,505 cases, 12.42%) and ‘respiratory, thoracic and mediastinal disorders’ (5,755 cases, 10.99%).

**Table 2:** Number of irAEs grouped by 18 tissue or organ disorders for FDA-approved ICIs

	Cemiplimab	Nivolumab	Pembrolizumab	Atezolizumab	Avelumab	Durvalumab	Ipilimumab
	Number/ratio	Number/ ratio	Number/ ratio	Number/ ratio	Number/ratio	Number/ ratio	Number/ratio
BL	49/0.078	4,336/0.051	2,975/0.057	2,070/0.097	163/0.064	604/0.075	438/0.054
CD	35/0.056	3,947/0.046	2,117/0.040	1,074/0.050	151/0.060	435/0.054	561/0.069
ELD	1/0.002	382/0.004	177/0.003	93/0.004	7/0.003	29/0.004	18/0.002
EnD	25/0.040	4,346/0.051	2,234/0.043	848/0.040	124/0.049	289/0.036	289/0.036
ED	8/0.013	1,664/0.019	919/0.018	269/0.013	27/0.011	108/0.013	78/0.010
GD	62/0.099	11,231/0.131	6,505/0.124	3,093/0.145	357/0.141	939/0.117	1,233/0.152
HD	50/0.080	4,666/0.055	3,009/0.057	1,405/0.066	133/0.053	463/0.058	553/0.068
ID	33/0.053	1,539/0.018	1,151/0.022	366/0.017	60/0.024	84/0.010	98/0.012
MnD	39/0.062	6,095/0.071	3,012/0.058	1,494/0.070	173/0.068	342/0.043	490/0.060
McD	30/0.048	5,544/0.065	3,280/0.063	1,029/0.048	145/0.057	418/0.052	335/0.041
NMU	36/0.057	10,113/0.118	6,764/0.129	1,121/0.053	153/0.060	1,008/0.126	1,781/0.220
NsD	69/0.110	7,168/0.084	4,278/0.082	1,990/0.093	243/0.096	627/0.078	559/0.069
PD	16/0.025	1,836/0.021	1,136/0.022	381/0.018	45/0.018	148/0.018	159/0.020
RuD	31/0.49	3,827/0.045	2,661/0.051	1,261/0.059	162/0.064	259/0.032	382/0.047
RB	3/0.005	259/0.003	205/0.004	95/0.004	6/0.002	20/0.002	14/0.002
RTM	73/0.116	9,317/0.109	5,755/0.110	2,429/0.114	277/0.110	1,526/0.190	849/0.105
SSD	40/0.064	6,631/0.078	4,414/0.084	1,362/0.064	168/0.066	439/0.055	1/0.000
VD	28/0.045	2,550/0.030	1,743/0.033	918/0.043	135/0.053	283/0.035	271/0.033
Total number cases	628	85,451	52,335	21,298	2,529	8,021	8,109

The three most common tissue or organ disorders after treatment with PD-L1 inhibitors were, for atezolizumab, ‘gastrointestinal disorders’ (3,093 cases, 14.52%), ‘respiratory, thoracic and mediastinal disorders’ (2429 cases, 11.40%), and ‘blood and lymphatic system disorders’ (2,070 cases, 9.72%) (Fig. 4 and Supplementary Figure S3B); for avelumab, ‘gastrointestinal disorders’ (357 cases, 14.12%), ‘respiratory, thoracic and mediastinal disorders’ (277 cases, 10.95%), and ‘nervous system disorders’ (243 cases, 9.61%); and for durvalumab, ‘respiratory, thoracic and mediastinal disorders’ (1,526 cases, 19.03%), ‘neoplasms benign, malignant and unspecified’ (1,008 cases, 12.57%) and ‘gastrointestinal disorders’ (939 cases, 11.71%).

For the CTLA-4 inhibitor ipilimumab, the three most common tissue or organ disorders were ‘neoplasms benign, malignant and unspecified’ (1,781 cases, 21.97%), ‘gastrointestinal disorders’ (1,233 cases, 15.21%) and ‘respiratory, thoracic and mediastinal disorders’ (849 cases, 10.47%) (Fig. 4 and Supplementary Figure S3C). In summary, ‘respiratory, thoracic and mediastinal disorders’ and ‘gastrointestinal disorders’ are the two major disorders caused by these seven ICIs.

### Tissue or organ disorders caused by FDA-approved ICIs vary widely by age group

**Fig. 5:**
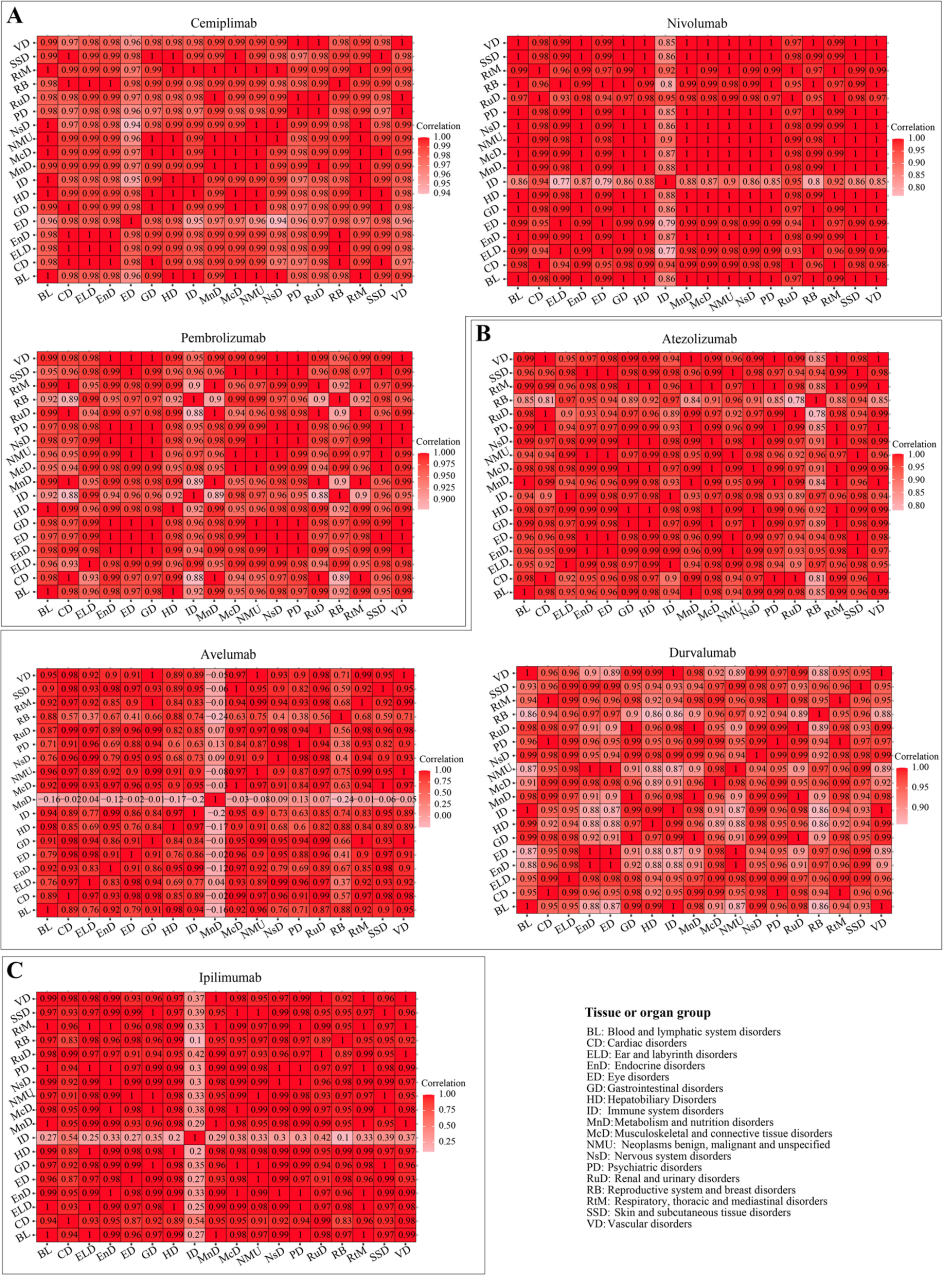
Correlation between irAEs and 18 tissue or organ disorders in different patient age groups. Correlations for ICIs targeting PD1, PDL1 and CTLA4 are shown in (**A**), (**B**) and (**C**), respectively

The proportions of tissue or organ disorders for these ICIs varied in different age groups (Supplementary Figure S3), shown in descending order of irAE rate in the 65–85 age group (left to right). We calculated the correlations between these tissue or organ disorders in patients treated with ICIs (Fig. 5 and Supplementary Table S1).

‘Nervous system disorders’ (17%) was the most common type of disorder caused by cemiplimab in patients with age 65–85 and was significantly correlated with ‘metabolism and nutrition disorders’ (Spearman value = 0.99, *P* = 0.004) and ‘psychiatric disorders’ (Spearman value = 0.99, *P* = 0.038). ‘Immune system disorders’ (15%) was the second most common disorder in patients aged 65–85 and was significantly correlated with ‘blood and lymphatic system disorders’ (Spearman value = 1, *P* < 0.0001), ‘endocrine disorders’ (Spearman value = 0.98, *P* = 0.02), ‘hepatobiliary disorders’ (Spearman value = 1, *P* = 0.002), and ‘musculoskeletal and connective tissue disorders’ (Spearman value = 0.99, *P* = 0.004). ‘Blood and lymphatic system disorders’ (14%) was the third most common disorder caused by cemiplimab in patients aged 65–85 and was significantly correlated with ‘endocrine disorders’ (Spearman value = 0.98, *P* = 0.009), ‘gastrointestinal disorders’ (Spearman value = 0.99, *P* = 0.001), ‘hepatobiliary disorders’ (Spearman value = 1, *P* = 0.004), ‘musculoskeletal and connective tissue disorders’ (Spearman value = 1, *P* < 0.0001) and ‘neoplasms benign, malignant and unspecified’ (Spearman value = 1, *P* = 0.027). The three most common disorders among patients aged 18–64 treated with cemiplimab were eye [(13%), and significantly correlated with ‘cardiac disorders’ (Spearman value = 0.98, *P* = 0.027)]; hepatobiliary [(10%), and significantly correlated with ‘blood and lymphatic system disorders’ (Spearman value = 1, *P* = 0.0004), ‘endocrine disorder’ (Spearman value = 0.99, *P* = 0.022), ‘immune system disorders’ (Spearman value = 1, *P* = 0.002) and ‘musculoskeletal and connective tissue disorders’ (Spearman value = 1, *P* = 0.014)]; and skin and subcutaneous tissue [(12%), and significantly correlated with ‘blood and lymphatic system disorders’ (Spearman value = 0.9, *P* = 0.012), ‘endocrine disorder’ (Spearman value = 0.99, *P* = 0.027), ‘gastrointestinal disorders’ (Spearman value = 0.99, *P* = 0.029), ‘immune system disorders’ (Spearman value = 0.96, P = 0.021), ‘musculoskeletal and connective tissue disorders’ (Spearman value = 1, *P* = 0.007) and ‘neoplasms benign, malignant and unspecified’ (Spearman value = 0.9, *P* = 0.003)].

For nivolumab and pembrolizumab, the three most common types of disorders among treated patients aged 65–85 were ‘cardiac disorders’, ‘renal and urinary disorders’ and ‘metabolism and nutrition disorders’, while ‘reproductive system and breast disorders’, ‘hepatobiliary disorders’ and ‘blood and lymphatic system disorders’ were the top 3 among patients aged 18–64; these were significantly correlated among these tissue or organ disorders (Spearman value > 0.8, *p* value < 0.05) (Supplementary Table S1).

Overall, our data show a highly varied influence of different ICIs on ‘reproductive system and breast disorders’. For ipilimumab patients (58 cases), breast pain was the most common AE among the reproductive system and breast disorders (17.2%), followed by scrotal edema (8.62%), female genital tract fistula (6.96%), pelvic pain (5.17%), and erectile dysfunction (5.17%). Among patients treated with atezolizumab (46 cases), prostatitis (15.2%) was the most common reproductive system/breast disorder followed by vaginal hemorrhage (13.04%), pelvic pain (13.04%), female genital tract fistula (13.04%) and erectile dysfunction (6.52%). For nivolumab patients (136 cases), pelvic pain (9.56%), breast pain (7.35%), vaginal hemorrhage (6.62%), erectile dysfunction (6.62%), and scrotal edema (4.41%) were the most common AEs among the reproductive system and breast disorders in cancer patients aged 18–64.

The three most common types of disorders among patients aged 65–85 treated with atezolizumab were ‘renal and urinary disorders’ (52%), ‘cardiac disorders’ (50%) and ‘metabolism and nutrition disorders’ (49%) (Figure S3B) and they were significantly correlated with other disorders (*P* value < 0.05). Among patients aged 18–64, the three most common disorders were ‘immune system disorders’ (43%), ‘ear and labyrinth disorders’ (42%), ‘reproductive system and breast disorders’ (41%) (a higher correlation among these disorders, except ‘immune system disorders’ (*P* = 0.065 > 0.05)).

For avelumab, the three most common types of disorders among treated patients aged 65–85 were ‘psychiatric disorder’ (71%, significantly correlated with other disorders except ‘metabolism and nutrition disorders’), ‘nervous system disorders’ (60%, significantly correlated with other disorders except ‘ear and labyrinth disorders’ and ‘metabolism and nutrition disorders’), ‘ear and labyrinth disorders’ (57%, significantly correlated with ‘immune system disorders’ and ‘neoplasms benign, malignant and unspecified’), while ‘reproductive system and breast disorders’ (67%, significantly correlated with ‘blood and lymphatic system disorders’, ‘hepatobiliary disorders’ and ‘psychiatric disorders’), ‘hepatobiliary disorders’ (42%, significantly correlated with other disorders expect ‘metabolism and nutrition disorders’) and ‘blood and lymphatic system disorders’ (42%, significantly correlated with other disorders except ‘ear and labyrinth disorders’) were the three most common disorders in patients aged 18–64.

Among patients aged 65–85 treated with durvalumab, the three most common types of disorders were ‘metabolism and nutrition disorders’ (49%, significantly correlated with other disorders except ‘psychiatric disorders’), ‘renal and urinary disorders’ (48%, significantly correlated with other disorders except ‘ear and labyrinth disorders’ and ‘psychiatric disorders’) and ‘cardiac disorders’ (47%, significantly correlated with other disorders except ‘ear and labyrinth disorders’ and ‘psychiatric disorders’).

For ipilimumab, ‘cardiac disorders’ (49%), ‘renal and urinary disorders’ (47%) and ‘vascular disorders (44%) were the three most common types of disorders (significantly correlated with other disorders expect ‘immune system disorders’) in patients with age 65–85; and ‘reproductive system and breast disorders’ (52%), ‘hepatobiliary disorders’ (47%) and ‘ear and labyrinth disorders (45%) were the three most common disorders (significantly correlated with other disorders expect ‘immune system disorders’) in patients aged 18–64.

Taken together, ‘cardiac disorders’ were the major type of disorder caused by these seven drugs among patients aged 65–85 and ‘reproductive system and breast disorders’ were the main type of disorder among patients aged 18–64.

### ‘Respiratory, thoracic and mediastinal disorders’ is the major type of disorder caused by FDA-approved ICIs in male patients, and ‘reproductive system and breast disorders’ in female patients

**Fig. 6:**
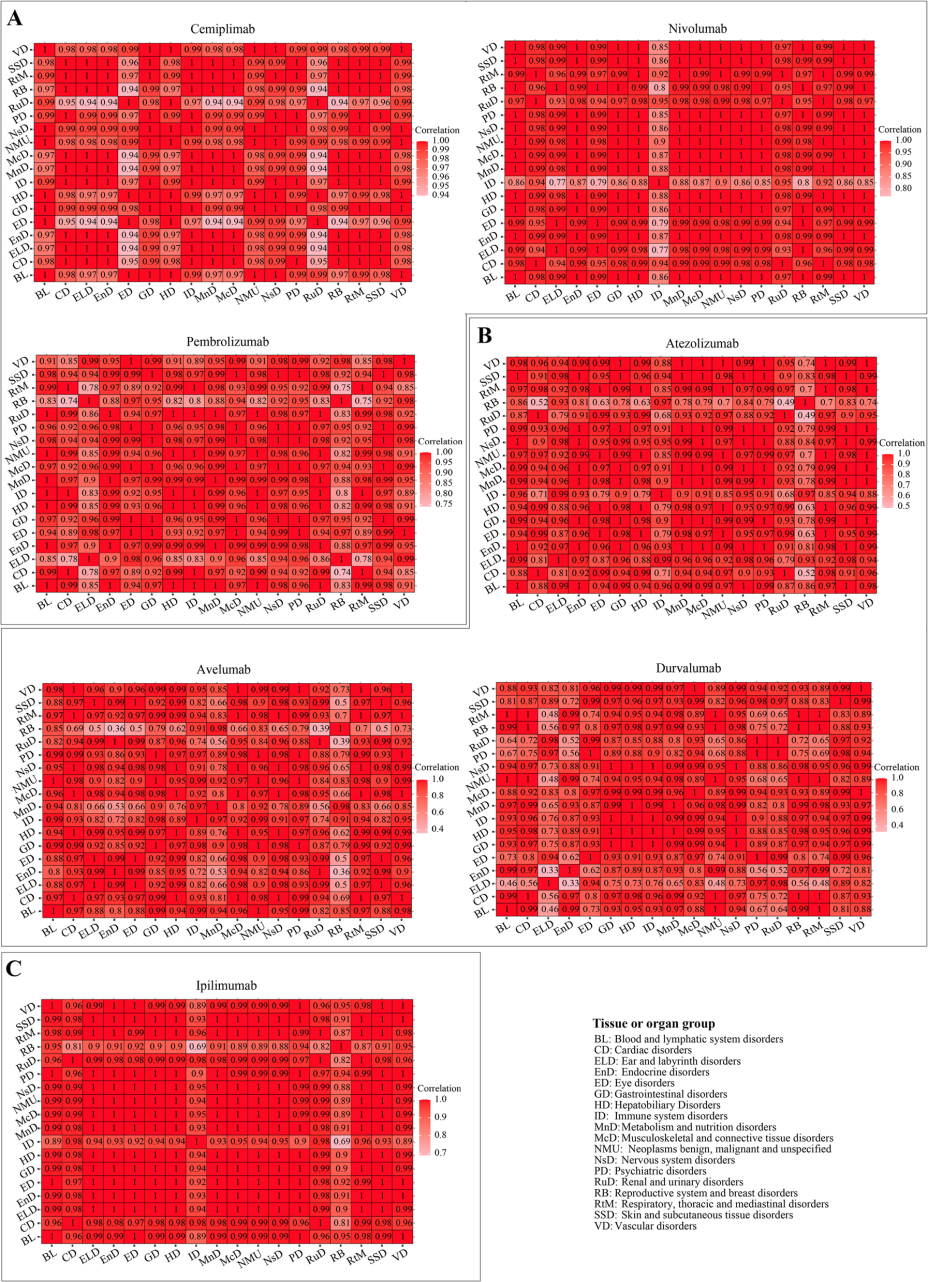
Correlation between irAEs and 18 tissue or organ disorders in different patient sex groups Correlations for ICIs targeting PD1, PDL1 and CTLA4 are shown in (**A**), (**B**) and (**C**), respectively

Next, we determined proportion of serious irAEs among total irAEs grouped by 18 tissue or organ disorders and associated with patient sex. (Supplementary Figure S4). Correlations between these disorders were also calculated (Fig. 6 and Table 3). ‘Renal and urinary disorders’ was one of the three most common types of disorders in male patients cause by PD-1 inhibitors, and was significantly correlated with ‘cardiac disorders’, ‘endocrine disorders’, ‘hepatobiliary disorders’ and ‘nervous system disorders’ in patients treated with nivolumab, and significantly correlated with ‘blood and lymphatic system disorders’ and ‘endocrine disorders’ in patients treated with pembrolizumab (Fig. 6A). ‘Reproductive system and breast disorders’ was one of the three most common types of disorders (all correlation *P* value > 0.05) in females treated with nivolumab (37%, none) and pembrolizumab (52%, none). ‘Renal and urinary disorders’ (59%, none) was the most common disorder caused by atezolizumab in males and was significantly correlated with ‘cardiac disorders’ (*P* = 0.021), while ‘reproductive system and breast disorders’ (60%, *P* > 0.05) was the most common disorder in females (Fig. 6B). The three most common disorders caused by avelumab were ‘ear and labyrinth disorders’ (71%, significantly correlated with ‘eye disorder’), ‘respiratory, thoracic and mediastinal’ (64%, significantly correlated with ‘eye disorder’) and ‘vascular disorders’ (64%, significantly correlated with ‘nervous system disorder, *P* = 0.049’) in males respectively. ‘Reproductive system and breast disorders’ (50%, none), ‘metabolism and nutrition disorders’ (42%, none) and ‘immune system disorders’ (35%, none) were the three most common disorders in females (no correlation with other disorders, *P* > 0.05). Among patients treated with durvalumab, ‘blood and lymphatic system disorders’ (64%, significantly correlated with ‘cardiac disorders’, ‘metabolism and nutrition disorders’ and ‘reproductive system and breast disorders’), ‘respiratory, thoracic and mediastinal’ (61%, significantly correlated with ‘eye disorders’, ‘musculoskeletal and connective tissue disorders’, and ‘nervous system disorders’), and ‘reproductive system and breast disorders’ (60%, significantly correlated with ‘blood and lymphatic system disorders’ and ‘cardiac disorders’) were the three most common disorders in male patients, while ‘ear and labyrinth’ (52%, significantly correlated with ‘gastrointestinal disorders’), ‘psychiatric disorders’ (47%, none), and ‘renal and urinary disorders’ (46%, none) were the three most common disorders in females.

**Table 3:** Number of irAEs grouped by 18 tissue or organ disorders in different patient sex groups

	Cemiplimab Number/ratio		Nivolumab Number/ratio		Pembrolizumab Number/ratio		Atezolizumab Number/ratio		Avelumab Number/ratio		Durvalumab Number/ratio		Ipilimumab Number/ratio	
	Male	Female	Male	Female	Male	Female	Male	Female	Male	Female	Male	Female	Male	Female
BL	0/0.00	10/0.20	1,485/0.34	2,468/0.57	1,093/0.27	1,681/0.57	902/0.44	979/0.47	53/0.33	92/0.56	157/0.26	385/0.64	673/0.37	993/0.55
CD	1/0.03	2/0.06	1,111/0.28	2,497/0.63	695/0.33	1,283/0.61	344/0.32	599/0.56	41/0.27	82/0.54	132/0.30	243/0.56	479/0.29	1,035/0.62
ELD	0/0.00	0/0.00	149/0.39	222/0.58	89/0.50	77/0.44	47/0.51	44/0.47	1/0.14	5/0.71	15/0.52	11/0.38	67/0.34	122/0.62
EnD	0/0.00	0/0.00	1,458/0.34	2,513/0.58	856/0.38	1,132/0.51	327/0.39	366/0.43	32/0.26	56/0.45	80/0.28	140/0.48	1,071/0.34	1,715/0.55
ED	0/0.00	2/0.25	620/0.37	912/0.58	421/0.46	446/0.49	94/0.35	136/0.51	6/0.22	15/0.56	46/0.43	46/0.43	321/0.35	511/0.56
GD	0/0.00	9/0.15	3,849/0.34	6,497/0.55	2,834/0.44	3,267/0.50	1,235/0.40	1,522/0.49	107/0.30	203/0.57	345/0.37	501/0.53	2,555/0.34	4,201/0.56
HD	2/0.4	11/0.22	1,539/0.33	2,686/0.58	1,092/0.36	1,696/0.56	494/0.35	727/0.52	33/0.25	75/0.56	164/0.35	234/0.51	881/0.34	1,491/0.57
ID	4/0.12	6/0.18	420/0.27	704/0.58	403/0.36	596/0.52	172/0.47	145/0.40	21/0.35	29/0.48	30/0.36	39/0.46	214/0.29	346/0.47
MnD	3/0.08	3/0.08	1,994/0.33	3,737/0.46	1,183/0.39	1,639/0.54	600/0.40	746/0.50	72/0.42	87/0.50	114/0.33	192/0.56	1,096/0.34	1,924/0.61
McD	3/0.10	3/0.10	1,863/0.34	3,252/0.61	1,410/0.43	1,634/0.50	411/0.40	494/0.48	40/0.28	74/0.51	163/0.39	202/0.48	741/0.33	1,304/0.58
NMU	1/0.03	7/0.19	3,225/0.32	6,146/0.59	2,466/0.36	3,816/0.56	424/0.38	593/0.53	48/0.31	91/0.59	281/0.28	582/0.58	1,338/0.33	2,293/0.57
NsD	3/0.04	12/0.17	2,439/0.34	4,238/0.61	1,797/0.42	2,215/0.52	847/0.43	941/0.47	58/0.24	148/0.61	225/0.36	332/0.53	1,145/0.33	2,089/0.60
PD	2/0.13	3/0.19	635/0.35	1,132/0.59	506/0.45	584/0.51	160/0.42	198/0.52	13/0.29	28/0.62	70/0.47	65/0.44	321/0.37	513/0.59
RuD	0/0.00	8/0.26	1,023/0.27	2,494/0.62	990/0.37	1,525/0.57	391/0.31	739/0.59	28/0.17	97/0.60	120/0.46	108/0.42	504/0.28	1,150/0.65
RB	0/0.00	0/0.00	97/0.37	152/0.65	106/0.52	87/0.42	57/0.60	34/0.36	3/0.50	3/0.50	6/0.30	12/0.60	49/0.47	52/0.50
RTM	5/0.07	10/0.14	2,782/0.30	5,763/0.59	1,900/0.33	3,454/0.60	901/0.37	1,225/0.50	69/0.64	178/0.64	416/0.27	934/0.61	1,059/0.32	2,051/0.61
SSD	3/0.08	5/0.13	2,244/0.34	3,842/0.58	1,831/0.41	2,214/0.50	537/0.39	593/0.44	40/0.52	88/0.52	177/0.40	196/0.45	1,189/0.34	1,979/0.57
VD	2/0.07	6/0.21	885/0.35	1,522/0.60	815/0.47	802/0.46	355/0.39	459/0.50	35/0.26	86/0.64	112/0.40	144/0.51	444/0.37	673/0.57

The most common disorders among male patients treated with ipilimumab were ‘renal and urinary disorders’ (65%, significantly correlated with ‘cardiac disorders’, ‘hepatobiliary disorders’ and ‘musculoskeletal and connective tissue disorders’), ‘ear and labyrinth disorders’ (62%, none) and ‘cardiac disorders’ (62%, significantly correlated with ‘hepatobiliary disorders’, ‘musculoskeletal and connective tissue disorders’ and ‘renal and urinary disorders’), ‘respiratory, thoracic and mediastinal disorders’ (61%, significantly correlated with ‘cardiac disorders’, ‘endocrine disorders’, ‘hepatobiliary disorders’ and ‘musculoskeletal and connective tissue disorders’, etc.). Among female patients treated with ipilimumab, the most common disorders were ‘reproductive system and breast disorders’ (47%, significantly correlated with ‘metabolism and nutrition disorders’, ‘nervous system disorders’, etc.), ‘blood and lymphatic system disorders’ (37%, significantly correlated with ‘metabolism and nutrition disorders’), ‘vascular disorders’ (37%, significantly correlated with ‘blood and lymphatic system disorders’ and ‘metabolism and nutrition disorders’) and ‘psychiatric disorders’ (37%, significantly correlated with ‘endocrine disorders’, ‘hepatobiliary disorders’, etc.), and ‘eye disorders’ (35%, significantly correlated with ‘endocrine disorders’, ‘hepatobiliary disorders’, etc.). In summary, ‘respiratory, thoracic and mediastinal disorders’ was the major type of disorder caused by these ICIs in male patients, and ‘reproductive system and breast disorders’ in female patients.

### The molecular targets of FDA-approved ICIs are highly expressed in human lung

**Fig. 7:**
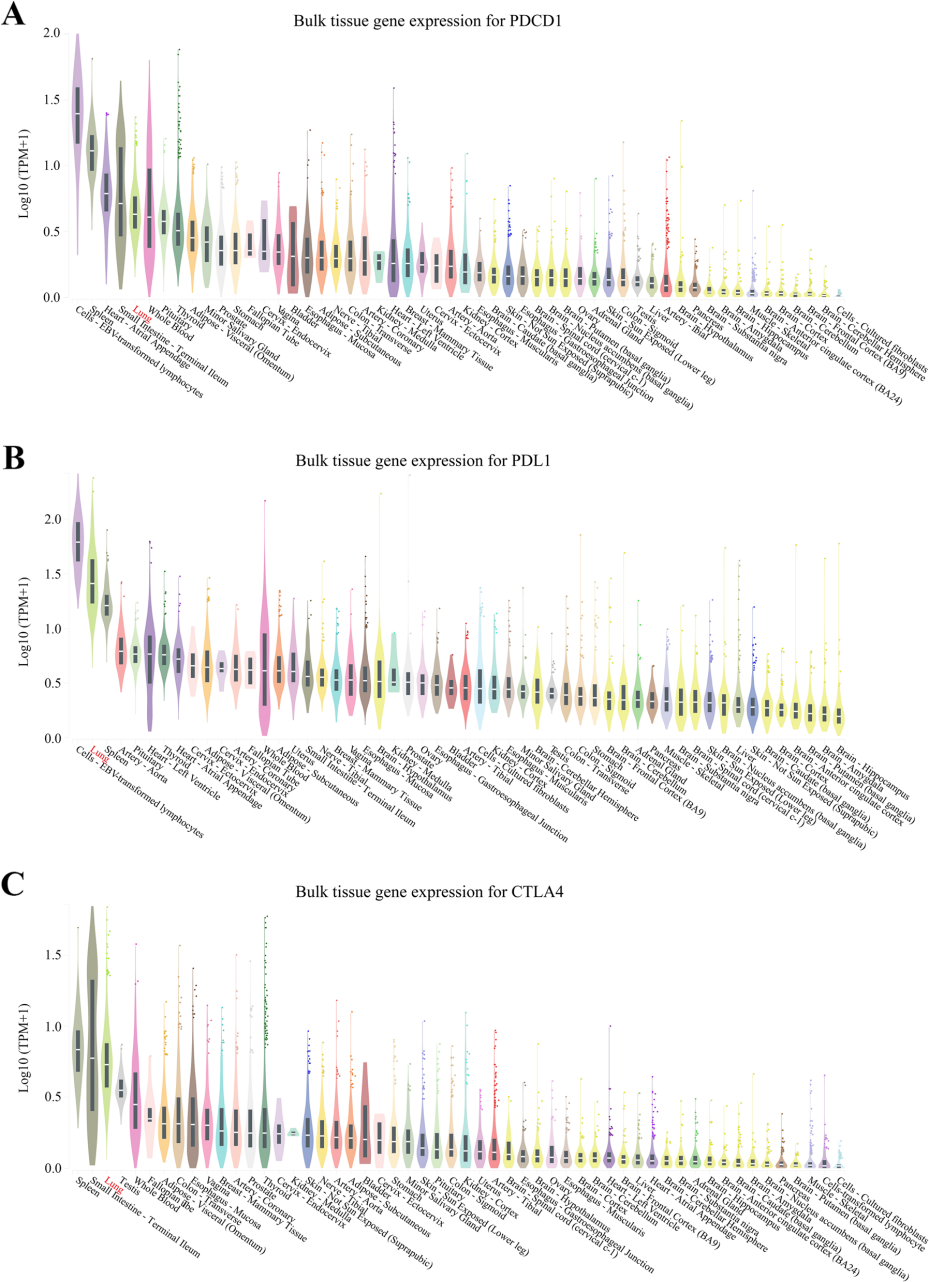
Expression of PDCD1 (encoding PD1), CD274 (encoding PD-L1) and CLTA4 in human tissues (including female and male). Tissue-specific gene expression for PDCD1, CD274 and CTLA4 are shown in (**A**), (**B**) and (**C**), respectively. These three genes are highly expressed in lung tissues regardless of sex

To better understand the effects of the seven drugs on tissue or organ disorders, expression levels of the PD-1, PD-L1, and CTLA-4 genes were analyzed in 30 human (male and female) tissues or organs, such as brain, adipose tissues, adrenal gland, bladder, blood vessel, breast, heart, kidney, liver, lung, ovary, spleen, uterus, etc., via the GTEx Portal dataset. The five tissues or organs with highest expression of the PD-1 gene were cells-EBV-transformed lymphocytes (median TPM value: male = 25.22, female = 22.57), spleen (median TPM value: male = 12.17, female = 11.40), heart-atrial appendage (median TPM value: male = 5.12, female = 5.14), small intestine-terminal ileum (median TPM value: male = 3.76, female = 5.01) and lung (median TPM value: male = 3.33, female = 3.16) (Fig. 7A). The five tissues or organs with highest expression of the PD-L1 gene were cells-EBV-transformed lymphocytes (median TPM value: male 66.74, female = 66.74), lung (median TPM value: male = 24.77, female = 23.88), spleen (median TPM value: male = 14.21, female = 15.29), artery (median TPM value: male = 5.21, female = 4.896) and pituitary (median TPM value: male = 4.66, female = 5.02) (Fig. 7B). The five tissues or organs with highest expression of the CTLA-4 gene were spleen (median TPM value: male = 5.60, female = 6.19), small intestine-terminal ileum (median TPM value: male = 4.56, female = 6.63), lung (median TPM value: male = 4.39, female = 4.13), testis (median TPM value: male = 2.53), whole blood (median TPM value: male = 1.78, female = 1.80) (Fig. 7C). In summary, the PD-1, PD-L1 and CTLA-4 genes were highly expressed in lungs in both males and females.

## Discussion

Cancer immunotherapy attempts to boost the body’s own defense mechanism to kill cancer cells and defeat cancer. Over the past few decades, immunotherapy with T cell checkpoint inhibitors has promised to revolutionize cancer therapy [3]. ICIs have offered new hope for cancer patients, especially for those with immunoactive tumors classified as “hot tumors” [20]. However, a major limitation of these therapies is that they are effective in only a subset of patients. Furthermore, the use of ICIs involves a series of related complications, namely, irAEs [21–23]. Recent research data revealed that up to 69% of ICI-treated patients develop acute or short-term AEs (13% of which are severe or fatal) and up to 43% of ICI patients display chronic or long-term (lasting three month or longer) AEs [21, 24]. It is difficult to identify the current evidence in the literature regarding risk factors or biomarkers for the whole category of ICIs as studies are typically either disease-specific (e.g., lung cancer or melanoma), or ICI drug-specific (e.g., pembrolizumab, ipilimumab), or irAE-specific (e.g., pneumonia or gastritis) [25]. It is extremely important to determine whether the assessment of ICI toxicity is needed to predict the occurrence of irAEs or provide early treatment that can modulate the immune system to obtain lasting antitumor effects [26–29]. As a new class of targeted anticancer drugs, ICIs target the immune tolerance pathways of tumor cells, such as PD-1, PD-L1 and CTLA-4, to kill tumor cells [30]. Chemokines and their receptors exert essential functions in all aspects of immune processes involved in physiology (hematopoiesis, immune defense and tissue health) and pathophysiology (chronic inflammation, allergy, and cancer), suggesting that irAEs may limit the use of chemokine-based reagents including ICIs in cancer treatment [3]. There is a clear need to understand the pattern of drug response and toxicity for ICIs. In this study, we focused on interrogating irAEs associated with treatment with seven FDA-approved ICIs. These included three PD-1 inhibitors (cemiplimab, nivolumab and pembrolizumab), three PD-L1 inhibitors (atezolizumab, avelumab and durvalumab) and one CTLA-4 inhibitor (ipilimumab).

Treatment of ICIs may cause the acute occurrence or the toxicity of any organ system, producing clinical AEs, which have been widely concerned [31–34]. There is an urgent need to develop reliable toxicity diagnosis and management methods to meet clinical needs. Few studies have determined tissue or organ-specific irAEs as they are mainly a discrete toxicity caused by the nonspecific activation of the immune system, reversible and easily overlooked [3]. Our data indicated that respiratory and gastrointestinal system toxicity is a common irAE in patients treated with these seven FDA-approved ICIs. It appears that lung cancer patients receiving ICIs are prone to serious irAEs. There are several reasons for this. Firstly, from clinical and research data [35–39], ICIs act as first or second-line treatment for lung cancer, and thus the numbers of lung cancer patients treated with ICIs are higher than in other tumor types. Our data also reveals respiratory system disorders are one of the three most common disorders among irAEs in lung cancer patients with ICIs. For instance, cemiplimab, as a new tumor immunotherapy agent showing anti-tumor activity and an acceptable safety profile, has been reported to improve the overall survival (OS) and progression-free survival (PFS) in advanced non-small-cell lung cancer (NSCLC) [39, 40]. However, cemiplimab treatment-related irAEs occur in 50% of NSCLC patients, and are more serious than in other tumors [29, 41]. Nivolumab is a second-line treatment for lung cancer [35], and is well tolerated in most patients yet has a wide range of irAEs because of its unique toxicity [42]. Pembrolizumab is a first-line monotherapy ICI improving OS and PFS in lung cancer patients, but grade 3 or worse treatment-related irAEs have occurred [15]. Our data also showed the number and proportion of serious irAEs in lung cancer are higher than in other types of cancers. Pembrolizumab and atezolizumab not only lead to lobular hepatitis in patients, but also result in sclerosing cholangitis, lymphocyte duct damage and granulomatous hepatitis. These agents can interact to cause impaired cellular functions such as CD8 ( +) lymphocytes and macrophages [15]. For avelumab, despite this drug showing good clinical results and an acceptable safety profile in solid tumor treatment, severe irAEs occur [43, 44], with respiratory disorders ranking second. ‘Respiratory, thoracic and mediastinal disorders’ is the leading type of disorder caused by durvalumab. Despite durvalumab showing high efficacy in the treatment of lung cancer, this drug can cause irAEs such as interstitial lung disease [38, 45], leading to discontinuation. The three-year OS of nivolumab plus ipilimumab (58%) is higher than that with ipilimumab (34%) [14, 37], and acute renal failure, diarrhea, hepatotoxicity, hepatitis, pneumonia, sepsis with acute renal insufficiency and thrombocytopenia are common phenomenon with treatment with these two ICIs [46]. Taken together, respiratory and gastrointestinal system toxicity is among the most common types of irAEs associated with the seven ICIs examined in this study.

The human immune system function declines annually with age and disorders among different immune system components are manifest by an enhanced response to autoantigens and decreased defense against microbes and cancer. Signs of "immune system aging" in humans may reduce the safety and efficacy of immune system-based treatment strategies or approaches and may lead to the occurrence of cancer and increased respiratory disease [47–50]. Huang et al. reported that older patients displayed a higher percentage of pulmonary toxicity when treated with ICIs (anti-PD-1/L1) [51], while another study found that older patients better tolerate treatment with ICIs [52, 53]. However, information about the irAEs generated by ICIs, and ICIs in older patients is still relatively limited. In this study, we analyzed the irAEs of seven ICIs across different age groups, including ages 18–64, 65–85 and > 85 years, and focused on irAEs among patients aged 65–85. ‘Renal and urinary disorders’ and ‘cardiac disorders’ were the most common types of disorders with these seven ICIs in patients aged 65–85 years. Compared with older patients, ‘reproductive system and breast disorders’ were the main irAEs caused by ICIs in patients aged 18–64. Age-related changes in the immune system may affect the efficacy and toxicity of ICI drugs [51, 54]. Here we demonstrate that patients of age 65–85 are more susceptible to ICI-related renal and urologic toxicity, while those of age 18–64 have more reproductive toxicity.

ICIs appear to more commonly produce respiratory and urinary system toxicity in male patients and reproductive system toxicity in female patients. The immune systems differ significantly between men and women after adolescence, which have profound implications for health and disease [55–57]. which are also manifest in the response to ICIs [58]. As immune function changes with age, adult women demonstrate greater inflammation and responsiveness than adult men [20, 50]. In general, sex differences in immune responses are more pronounced in young adults, which is also evident in older men and women [50]. In addition, older women are more likely to develop autoimmune diseases than older men, while older men are more prone to develop tumors than older women [50, 55, 57]. In a variety of immune cells, sex hormones can bind to specific receptors to achieve immune function [59, 60]. Chen et al. reported that men are more likely to develop ICI-associated renal toxicity with a longer median time of onset and poor prognosis [59]. Our data revealed that ICIs show greater toxicity in respiratory and urinary systems than in other tissues or organs in men, and more intense reproductive toxicity in women. PD-1, PD-L1 and CTLA-4 are slightly different in male and female tissues, therefore, insight into their pathogenesis and interactions may help in better development of immunotherapy strategies to promote clinical care of patients [49, 50]. Moreover, an accurate assessment of the effects of cancer immunotherapy in males and females, and the assessment of the applicability of various tumor models for predicting the sex-dependent success of specific immunotherapies, is crucial [59].

ICIs developed to target immune checkpoint proteins have been successfully used to treat patients with metastatic melanoma, HNC, and NSCLC [61–64]. A high percentage of cancer patients treated with ICIs has ‘respiratory, thoracic, and mediastinal disorders’, suggesting the dysfunction of patients’ lungs. The expression levels of immune checkpoint-coding genes may implicate their importance in the corresponding tissues, and inhibiting their expression may lead to functional disturbance of these tissues, which could be a causative factor for organ-specific irAEs. By analyzing the GTEx dataset, we found higher levels of genes encoding PD-1, PD-L1 and CTLA in human lung tissues than in other organs. This may explain why lung-irAEs occur more frequently in ICI-treated patients. On the other hand, emerging evidence supports the notion that irAEs may be reflective of mechanism-based autoimmune or inflammatory reactions towards the ICI. Previous reports have described that irAEs can prolong OS and PFS by about 6 or 3 months, respectively [65, 66]. For example, the presence of overall irAEs was significantly associated with longer OS in melanoma or NSCLC patients treated with nivolumab [67, 68] Recently, a retrospective study of 156 patients who were treated with ICIs compared 82 patients with irAEs with 74 patients with non-irAEs, and indicated that PFS and OS in the irAE group were significantly longer than those in the non-irAE group [69]. Judo et al. reported that only low grade irAEs, but not high grade irAEs, are associated with better responses to anti-PD-1 antibodies in non-melanoma patients [70]. These findings suggest that irAEs could be used to predict more favorable clinical outcomes of ICI therapy given that irAEs are managed appropriately. The effect of irAEs on PFS and OS is evident; however, lung-irAEs can occur at a later phase than non-lung-irAEs (skin, endocrine, digestive tract) and seemed not to prolong OS and PFS. To this end, understanding the general aspect of lung-irAE is a critical issue for cancer researchers.

Our data may differ from previously reported data for several reasons. Different data sources may be one cause of discrepancies. For example, Baggi’s study analyzed data from 131 advanced and metastatic cutaneous squamous cell carcinoma cases [41], while our study included 492 total irAE cases across 13 common tumors. Another important factor is the difference in statistical objects. The rate of grade 3–4 irAEs was 9.2%, while treatment-related irAEs were seen in 42.7% of the total patients in Baggi’s study [41], which is very similar to the data we reported. As for specific ICIs, the rates of grade 3–4 treatment emergent AEs and serious irAEs were 44% and 29%, respectively, in advanced cutaneous squamous cell carcinoma patients with cemiplimab treatment [40], while the rates of grade 3 and 4 irAEs were 13.8% and 73.2%, respectively, in small-cell lung cancer patients treated with nivolumab [71]. These studies were conducted at a single institution with higher or lower medical comorbidities and reported cancer disparities compared to the rest of the country or only for a single ICI. Our current study estimated irAEs of seven FDA-approved ICIs in the United States. For example, the rate of cemiplimab treatment-associated AEs in lung cancer patients was 50% of the total cancer cases reported to the FDA. Thus, the resulting data should be more applicable to preserving quality of life and avoiding or minimizing the risk of irAE-related fatal outcomes.

As the major consequence of ICI treatment, antitumor immunity is activated with infiltration of immune cells, including T cells, into tumors. Increased T cell diversity in response to ICI treatment could also be a sign of immune response to normal tissues. Oh et al. found that initial broadening in the repertoire of circulating T cells occurred within 2 weeks in patients with metastatic castration-resistant prostate cancer treated with a combination of ipilimumab and granulocyte-monocyte colony-stimulating factor, which significantly preceded irAE onset and was correlated with the development of irAEs [72]. To the best of our knowledge, there are few relevant studies reporting the potential association between innate immune cells and irAEs, which will be an interesting research direction in the future.

## Conclusions

In summary, our study focuses on the toxicity burden of seven FDA-approved ICIs in 13 common cancers and 18 tissues or organs of patients. ICIs have the highest probability of serious irAEs in patients with lung cancer, which may be associated with the respiratory toxicity of ICIs. Further, the tissue or organ toxicity of ICIs is age-and sex-specific. ICIs are associated with greater renal and urinary system toxicity among patients aged 65–85 years, reproductive toxicity in patients aged 18–64, respiratory and urinary system toxicity in males, and reproductive system toxicity in females. These differences in patients’ age and sex should be considered during ICI treatment. Further studies on the toxicity mechanism of ICIs are needed to provide more accurate basic research data for clinical practice.

## Supplementary information

**Additional file 1.**

**Additional file 2: Supplementary Figure S1**. Proportion of irAE outcomes for seven ICIs reported by the FDA annually (January 1, 2015 to June 30, 2022). The irAE cases for each drug were divided into seven outcome groups, including died, disabled, hospitalized, life-threatening, non-serious, required intervention and other outcomes. Percentages of hospitalized and life-threatening outcomes are indicated for each drug every year. Data for ICIs targeting PD-1, PD-L1 and CTLA4 are shown in (A), (B) and (C), respectively.** Supplementary Figure S2**. Proportion of serious irAEs among total irAEs for various types of cancer. Data for ICIs targeting PD-1, PD-L1 and CTLA4 are shown in (A), (B) and (C), respectively. **Supplementary Figure S3.** Proportion of serious irAEs among total irAEs grouped by 18 tissue or organ disorders and associated with patient age. Data for ICIs targeting PD-1, PD-L1 and CTLA4 are shown in (A), (B) and (C), respectively. **Supplementary Figure S4. **Proportion of serious irAEs among total irAEs grouped by 18 tissue or organ disorders and associated with patient sex. Data for ICIs targeting PD-1, PD-L1 and CTLA4 are shown in (A), (B) and (C), respectively.

## Data Availability

Data are available on reasonable request. All data relevant to the study are included in the article or uploaded as online supplemental information.

## Abbreviations

CTLA-4: Cytotoxic T lymphocyte-associated antigen
FAERS: FDA Adverse Events Reporting System
HNC: Head and neck cancer
irAEs: Immune-related adverse events
ICIs: Immune checkpoint inhibitors
NSCLC: Non-small cell lung cancer
OR: Odds ratio
OS: Overall survival
PD-1: Programmed death-1
PD-L1: Programmed death ligand-1
PFS: Progression-free survival
PT: MedDRA dictionary Preferred Term
TPM: Transcripts Per Kilobase Million
