# Supplementary material for: Patterns of toxicity burden for FDA-approved immune checkpoint inhibitors in the United States
Source: J Exp Clin Cancer Res. 2023 Jan 5;42:4. doi: 10.1186/s13046-022-02568-y (PMC9814433; doi:10.1186/s13046-022-02568-y)

**Supporting Information for**

**Patterns of toxicity burden for FDA-approved immune checkpoint inhibitors in the United States**

Fan Yang^1^, Chloe Shay^2^, Marin Abousaud^3^, Chris Tang^1^, Yamin Li^4^, Zhaohui Qin^5^, Nabil F. Saba^1^, Yong Teng^1*^

**Correspondence to:** Yong Teng, [yong.teng@emory.edu](mailto:yong.teng@emory.edu)

**This file includes:**

**Supplementary Figures and Figure Legends**

**Supplementary Figure S1. Proportion of irAE outcomes for seven ICIs reported by the FDA annually (January 1, 2015 to June 30, 2022).** The irAE cases for each drug were divided into seven outcome groups, including died, disabled, hospitalized, life-threatening, non-serious, required intervention and other outcomes. Percentages of hospitalized and life-threatening outcomes are indicated for each drug every year. Data for ICIs targeting PD-1, PD-L1 and CTLA4 are shown in (A), (B) and (C), respectively.


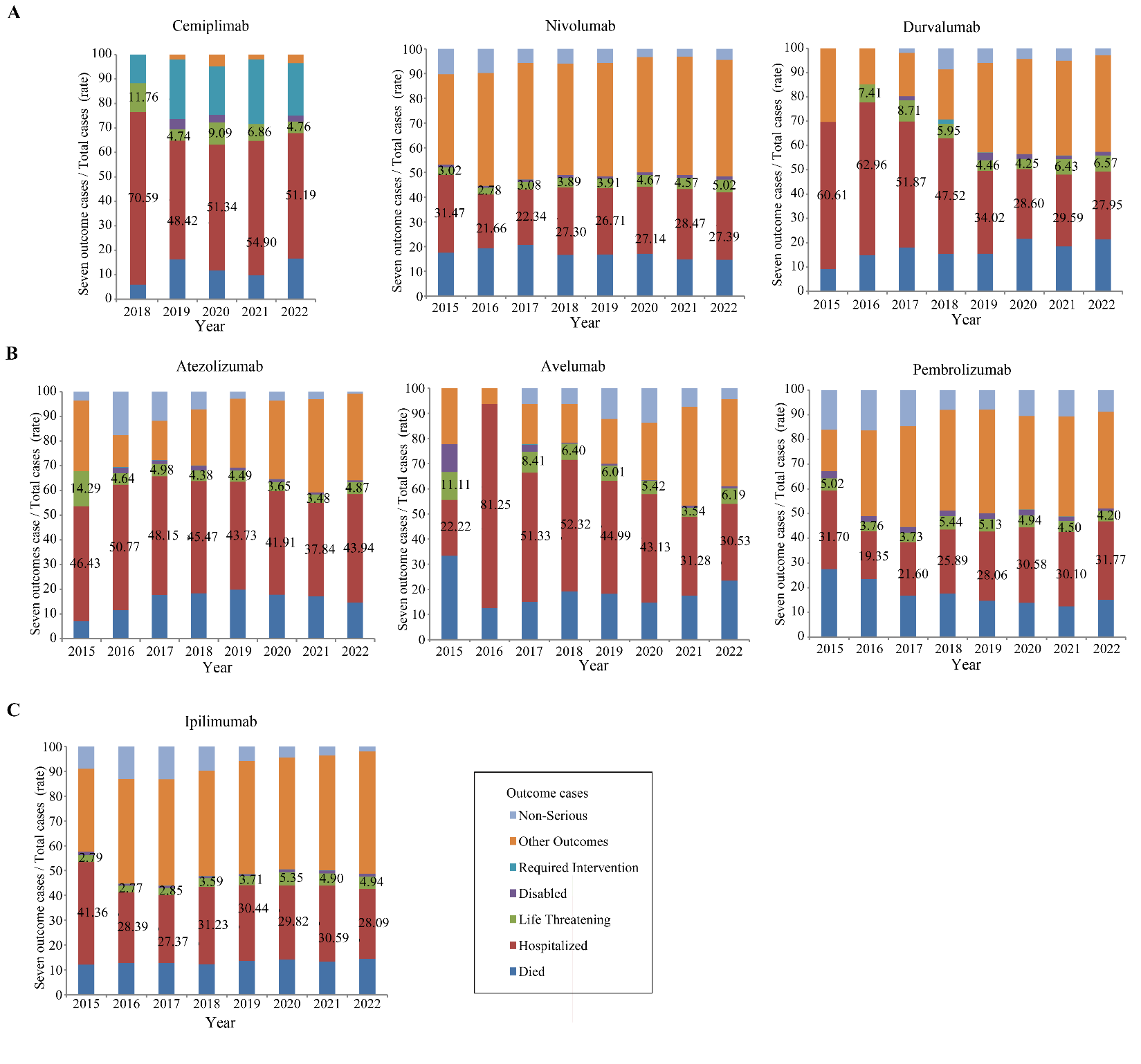


**Supplementary Figure S2. Proportion of serious irAEs among total irAEs for various types of cancer.** Data for ICIs targeting PD-1, PD-L1 and CTLA4 are shown in (A), (B) and (C), respectively.


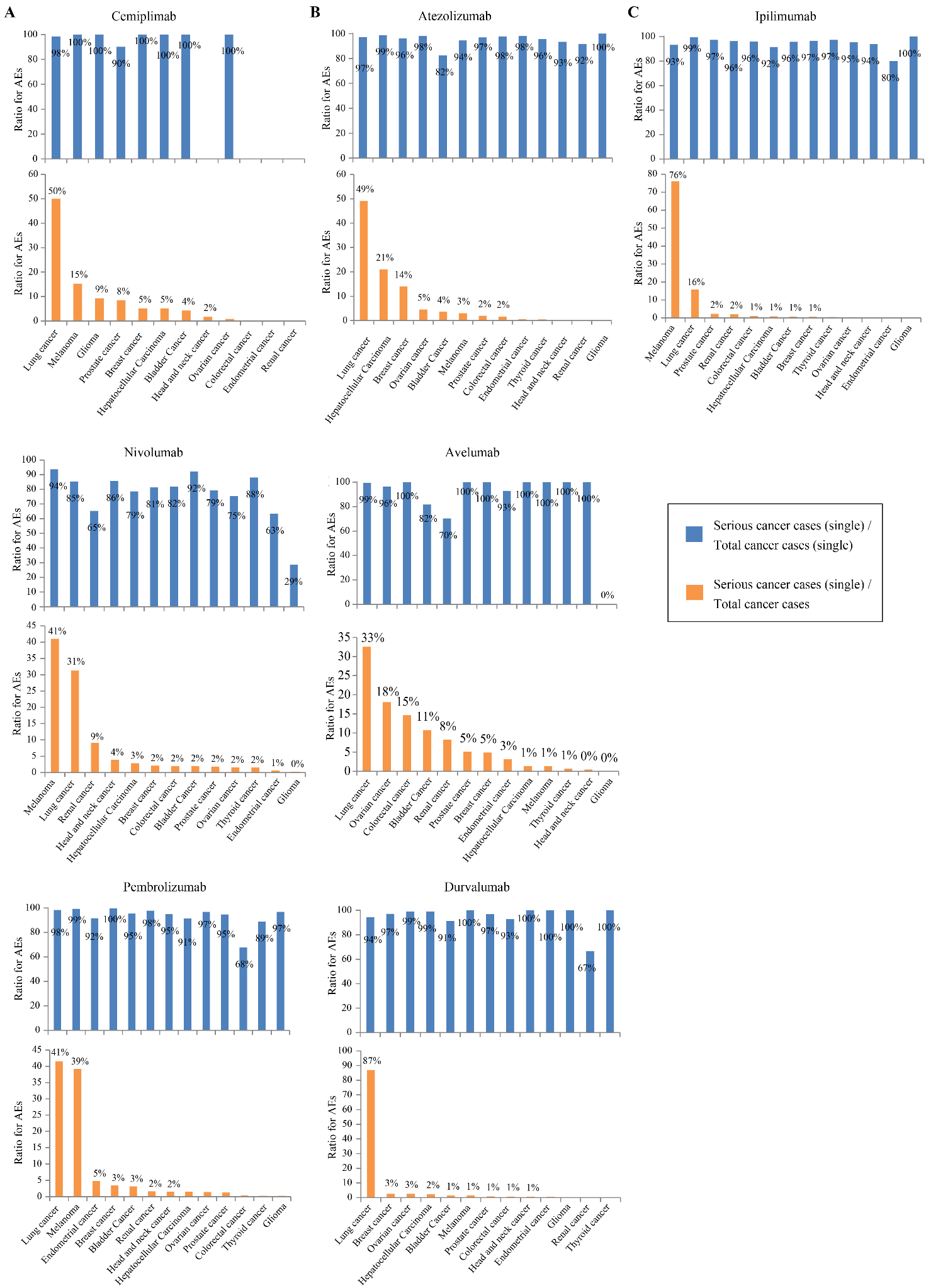


**Supplementary Figure S3. Proportion of serious irAEs among total irAEs grouped by 18 tissue or organ disorders and associated with patient age.** Data for ICIs targeting PD-1, PD-L1 and CTLA4 are shown in (A), (B) and (C), respectively.


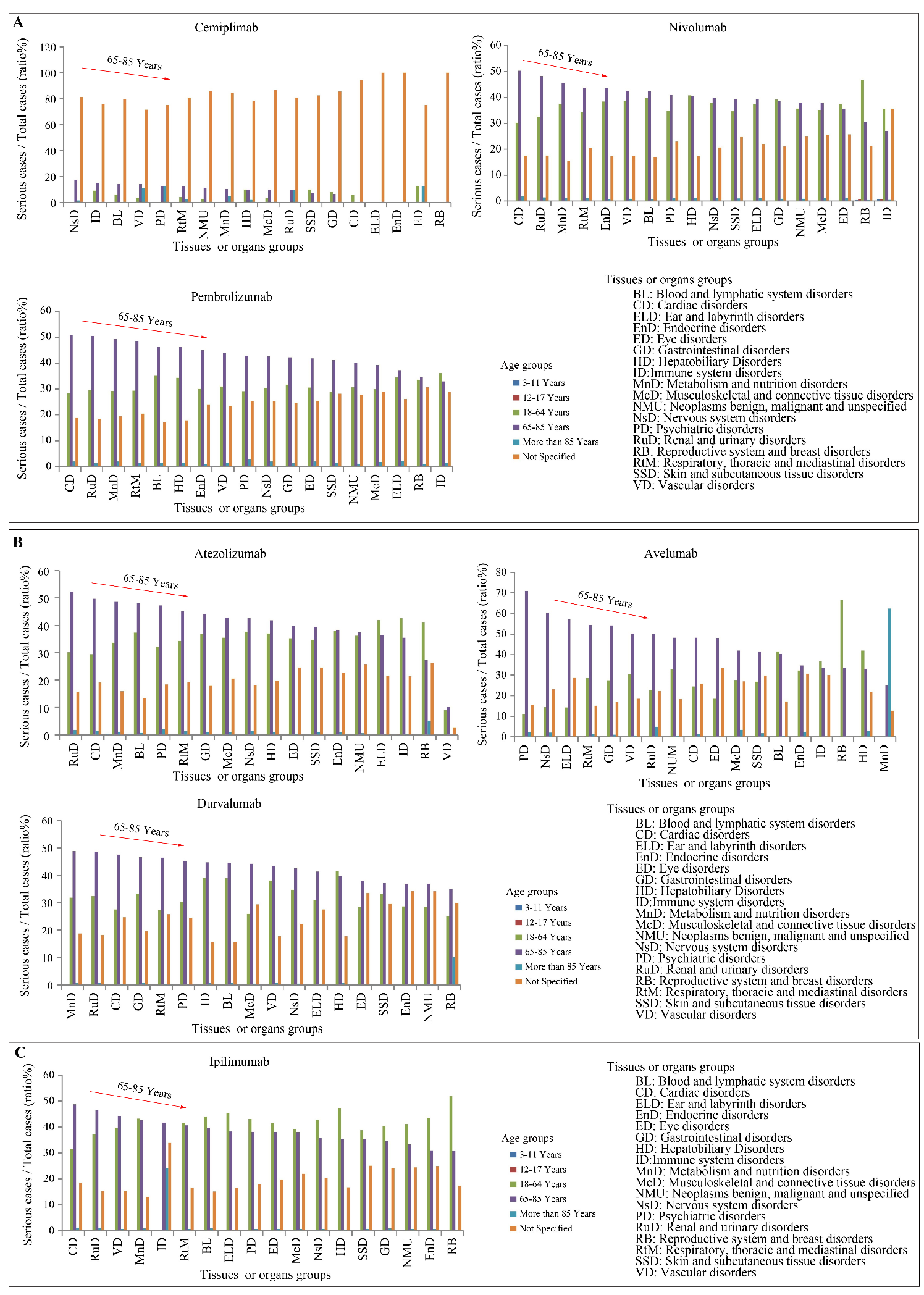


**Supplementary Figure S4. Proportion of serious irAEs among total irAEs grouped by 18 tissue or organ disorders and associated with patient sex.** Data for ICIs targeting PD-1, PD-L1 and CTLA4 are shown in (A), (B) and (C), respectively.


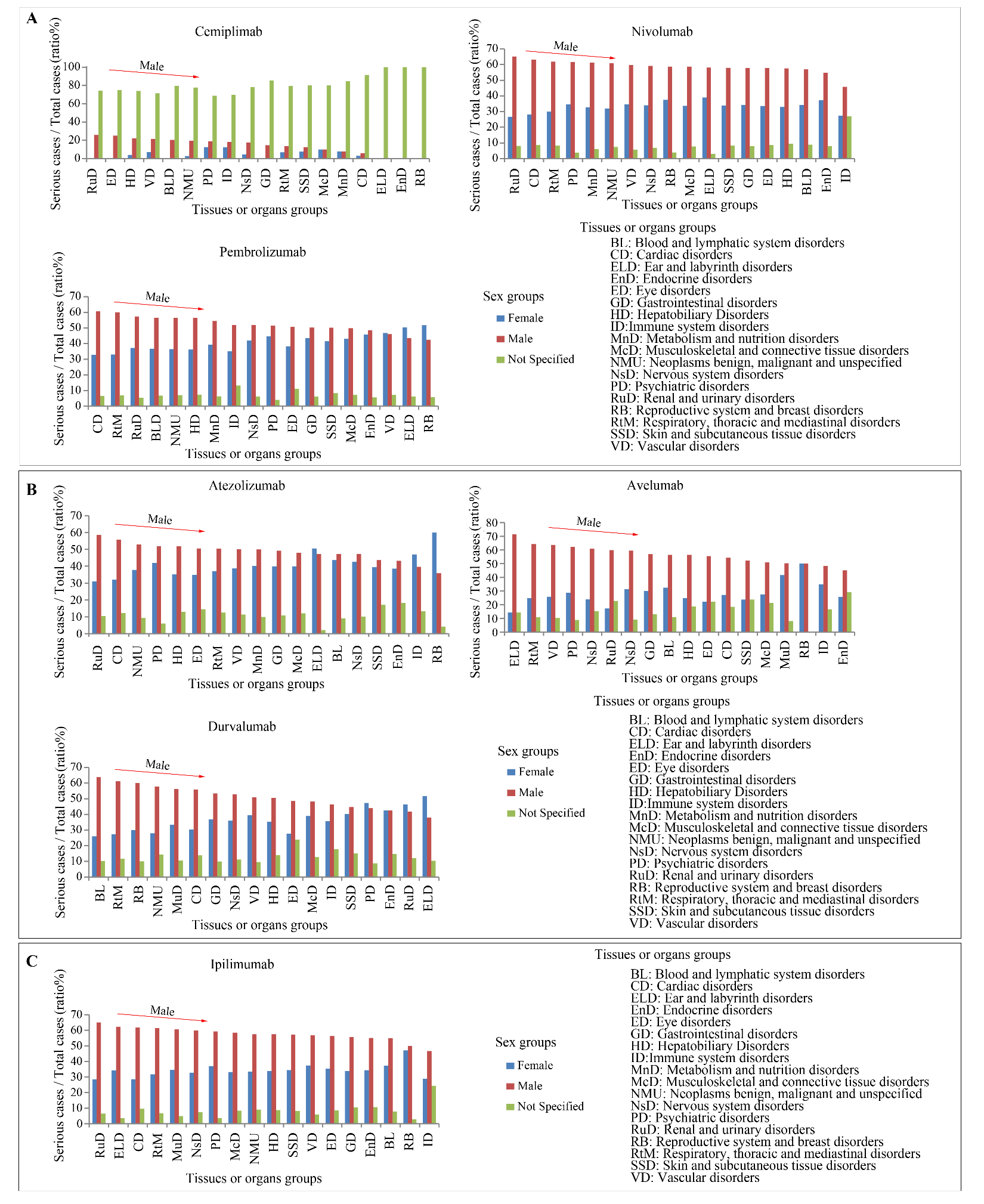

Supplement: Supplementary file 2 — Additional file 2: Supplementary Figure S1. Proportion of irAE outcomes for seven ICIs reported by the FDA annually (January 1, 2015 to June 30, 2022). The irAE cases for each drug were divided into seven outcome groups, including died, disabled, hospitalized, life-threatening, non-serious, required intervention and other outcomes. Percentages of hospitalized and life-threatening outcomes are indicated for each drug every year. Data for ICIs targeting PD-1, PD-L1 and CTLA4 are shown in (A), (B) and (C), respectively. Supplementary Figure S2. Proportion of serious irAEs among total irAEs for various types of cancer. Data for ICIs targeting PD-1, PD-L1 and CTLA4 are shown in (A), (B) and (C), respectively. Supplementary Figure S3. Proportion of serious irAEs among total irAEs grouped by 18 tissue or organ disorders and associated with patient age. Data for ICIs targeting PD-1, PD-L1 and CTLA4 are shown in (A), (B) and (C), respectively. Supplementary Figure S4. Proportion of serious irAEs among total irAEs grouped by 18 tissue or organ disorders and associated with patient sex. Data for ICIs targeting PD-1, PD-L1 and CTLA4 are shown in (A), (B) and (C), respectively. [file 13046_2022_2568_MOESM2_ESM.docx]
